# Educational fMRI: From the Lab to the Classroom

**DOI:** 10.3389/fpsyg.2019.02769

**Published:** 2019-12-06

**Authors:** Mohamed L. Seghier, Mohamed A. Fahim, Claudine Habak

**Affiliations:** Cognitive Neuroimaging Unit, Emirates College for Advanced Education (ECAE), Abu Dhabi, United Arab Emirates

**Keywords:** education, cognitive neuroscience, functional neuroimaging, translation, application, classroom, variability, reliability

## Abstract

Functional MRI (fMRI) findings hold many potential applications for education, and yet, the translation of fMRI findings to education has not flowed. Here, we address the types of fMRI that could better support applications of neuroscience to the classroom. This ‘educational fMRI’ comprises eight main challenges: (1) collecting artifact-free fMRI data in school-aged participants and in vulnerable young populations, (2) investigating heterogenous cohorts with wide variability in learning abilities and disabilities, (3) studying the brain under natural and ecological conditions, given that many practical topics of interest for education can be addressed only in ecological contexts, (4) depicting complex age-dependent associations of brain and behaviour with multi-modal imaging, (5) assessing changes in brain function related to developmental trajectories and instructional intervention with longitudinal designs, (6) providing system-level mechanistic explanations of brain function, so that useful individualized predictions about learning can be generated, (7) reporting negative findings, so that resources are not wasted on developing ineffective interventions, and (8) sharing data and creating large-scale longitudinal data repositories to ensure transparency and reproducibility of fMRI findings for education. These issues are of paramount importance to the development of optimal fMRI practices for educational applications.

## Introduction

For more than a century, many teaching methods have been developed and tested, by drawing on the implications of social, cognitive, and developmental psychology for educational practice. Typically, these teaching methods have been implemented in the educational setting without accounting for the workings of the ‘black box’ of the mind (Byrnes and Vu, 2015). There has been growing interest in how neuroscience can contribute to efficient learning in the classroom (Goswami, 2004; Petitto and Dunbar, 2009; Sousa, 2010; Frith, 2011; Sigman et al., 2014; Thomas et al., 2019a). For example, relevant neuroscience evidence can direct the implementation and design of efficient instruction methods, based on whether the instructions are compatible with how the brain processes information (Immordino-Yang and Gotlieb, 2017; Mayer, 2017; Thomas et al., 2019a). This requires educators to understand the neuroscience literature, and to be able to identify the most useful evidence for the educational context. A large proportion of the neuroscience literature comes from neuroimaging work, in particular findings obtained with functional MRI (fMRI). Even though fMRI is thought of as a single general tool, its application varies from one context to another: a better understanding of these applications would be useful for educators to better assess the types of fMRI that are most relevant to questions in education.

The amount of knowledge on how the brain works is growing exponentially, especially for learning-related functions, such as attention, affect, memory, decision-making and control, or for learning-related skills such as reading, writing, number processing, and problem solving (Peelen and Kastner, 2014; Nieder, 2016; Pessoa and McMenamin, 2017; Wandell and Le, 2017; Ng, 2018), and for their interactions with sleep, nutrition and physical activity (Khan and Hillman, 2014; Sigman et al., 2014). It makes sense to capitalize on this rising knowledge to optimize learning practice, especially when deciding on competing teaching models, or when seeking tailored support for individuals with learning difficulties (e.g. Neville et al., 2013). However, neuroscience findings are technical and complex, and they have not been framed within the context of education, so this extensive research has not yet found its impact on education.

Making sense of the wide-ranging fMRI findings, sometimes with inconsistent results across studies, can be challenging, which leads to a wider gap between neuroscience and education (Bowers, 2016). However, appropriate and transparent fMRI methods (Carp, 2012; Soares et al., 2016) that ensure reliable inferences (Bennett and Miller, 2010; Dubois and Adolphs, 2016) can be applied to educationally-relevant contexts. The apparent gap between education and neuroscience is typical of the growth process of any emerging field: evidence from neuroscience has faced resistance in other areas when evaluating its translational potential, with the classic example from clinical neuroscience. Here, we highlight the trends and best practices in fMRI, to maximize its potential implications for education; some of these practices are based on lessons learnt from clinical fMRI (Rosen, 2009).

The term ‘clinical fMRI’ was coined over two decades ago (Jezzard and Song, 1996; Thulborn et al., 1996) to highlight the potential implications of fMRI findings to the clinical setting (Detre and Floyd, 2001). Other specialized applications such as pharmacological fMRI (Jokeit et al., 2001; Harvey et al., 2018) and foetal- and newborn-fMRI (Hykin et al., 1999; Seghier et al., 2006) have also been developed for clinical applications. The potential use of clinical fMRI was initially met with resistance from clinicians, given the many potential pitfalls and limitations in making robust and reliable fMRI inferences for clinical practice (Desmond and Chen, 2002; Hennig et al., 2003; Haller and Bartsch, 2009; Dubois and Adolphs, 2016; Eklund et al., 2016; Turner, 2016; Roalf and Gur, 2017; Rizzolatti et al., 2018). The most frequent still-present critiques of fMRI by clinicians are six-fold: (1) fMRI is too slow, given its low temporal resolution and the inherent hemodynamic delays, (2) it only provides indirect measures (through neurovascular coupling) of neuronal activity, (3) it is preoccupied with the localization of brain “blobs” at arbitrary statistical thresholds with no insight into the biological mechanisms of brain function, (4) it is highly variable, with typically moderate intra-subject consistency and large between-subject variability, (5) it is notoriously difficult to conduct in vulnerable populations when participant cooperation (e.g. staying still and executing a task) is difficult, and (6) fMRI data is too complex, and it relies on sophisticated processing methods that may complicate result interpretation. Despite this, fMRI has become a useful tool in clinics, as a non-invasive option in addressing certain challenges, such as determining hemispheric dominance in patients, mapping vital brain functions before surgery, visualizing brain reorganization to support recovery, and localizing epileptic foci.

The relevance of clinical fMRI is expected to grow even more so, thanks to recent developments in cutting-edge techniques, and to a new paradigm shift in how fMRI can be applied: from standard static brain maps obtained with subtractive logic on data collected with block designs, to dynamic connectivity maps obtained while participants are doing ‘nothing’ (at rest) in the scanner. These developments are occurring at both conceptual and methodological levels (e.g. Turner, 2016), and they have opened new horizons in neuroscience, including systems neuroscience, precision neuroscience, and population neuroscience. However, despite these new developments, fMRI is still not seen as a tool of choice by researchers interested in educational topics because it is (1) an expensive technique with high running costs, (2) not portable, (3) not easy to use on young school-aged participants, (4) not flexible enough for large-scale studies or when simultaneous parallel acquisitions in multiple participants are of interest, (5) limited in terms of paradigm designs that can be delivered within a typical scanning environment, and (6) restricted to looking inside the brain while the participant is lying still in a tube, which is not ideal for the typical educational context with active students moving in class. Nevertheless, the unparalleled look inside the brain that fMRI can bring, outweighs these limitations; see for instance recent review about the potential of fMRI in extending our understanding of the neural basis of memory development (Ofen et al., 2019).

To frame fMRI within the educational context, it would make sense to define first the scope of educational neuroscience (or mind, brain and education). Educational neuroscience is a transdisciplinary domain concerned with two complementary questions: how neuroscience evidence can be used to optimize leaning and teaching, and how education can enhance intellectual abilities and change the brain. In a recent systematic thematic analysis of the literature over the last 30 years, three foundational pillars were identified for the field of educational neuroscience that include application, interdisciplinarity, and translation (Feiler and Stabio, 2018). According to some recent models, there are two pathways linking the application and translation of neuroscience to education, one direct pathway that considers the brain as a biological organ that needs to be in the optimal condition to learn, and an indirect pathway mediated by psychology as neuroscience shapes psychological theory and psychology influences education (Thomas et al., 2019a). Current neuroscience evidence is dominated by findings related to the indirect pathway. To increase impact through the direct pathway, neuroscience evidence needs to progress further towards system-level mechanistic explanations of learning, and to adopt a holistic view that contextualizes learning across multiple dimensions, encompassing wellbeing, social cognition, affective processing, nutritional factors, genetic factors, sleep, and exercise. This holistic approach involves the blending of diverse disciplines, research methodologies, and paradigms, which integrates multiple levels of analysis to form a multi-level yet coherent explanation for learning, and to inform educational practice and policy (Han et al., 2019). Here, the focus is to frame fMRI within the evolving and transdisciplinary interface of education and neuroscience.

Accordingly, there are many examples of how fMRI research has actually informed educational theories and practices, by providing, for example, biological explanations about brain-behaviour associations during learning and development, e.g. see recent work about the brain correlates of reading acquisition (Chyl et al., 2018; Takashima et al., 2019), and conceptual knowledge in STEM learning (Cetron et al., 2019). A review of this large body of literature can be found elsewhere (e.g. see Sousa, 2010; Butterworth et al., 2011; Sigman et al., 2014; Black et al., 2015; Ozernov-Palchik et al., 2016; Immordino-Yang and Gotlieb, 2017; Thomas et al., 2019a). The current review aims to address a different question: what type of fMRI is most useful for educational purposes? The answer to this question invites this “educational fMRI” to embrace new developments and emerging trends that are taking place in the field of functional neuroimaging and brain mapping. Specifically, this educational fMRI should be concerned with: (1) scanning young school-aged populations, (2) investigating samples with wide variability in learning abilities and disabilities, (3) studying the brain under natural and ecological conditions, (4) multi-modal imaging of brain-behaviour associations, (5) assessing developmental or instruction-induced changes in brain function with longitudinal designs, (6) providing system-level mechanistic explanations of brain function, (7) reporting (unbiased) negative findings, and (8) sharing data and creating large-scale longitudinal databases. A comprehensive list of all available strategies and practices in the current literature is beyond the scope of this review: our goal is to highlight that many ingenious solutions have been proposed in the literature, and that the field is changing rapidly, with many strategies and solutions becoming available to researchers in the near future, despite some of the arduous challenges addressed in this review. Although the abovementioned points may also concern the application of fMRI in other fields, they are discussed here in terms of existing challenges, new opportunities and best practices for fMRI in education.

### Scanning Young School-Aged Participants

Educational fMRI is mainly concerned with studying brain function in young populations, as opposed to the dominant representation of adults in the current fMRI literature. However, carrying-out fMRI in children is generally recognized as more challenging than fMRI in adults. There are many difficulties when scanning children in terms of (1) recruitment, (2) ensuring compliance and participant cooperation, (3) stringent ethical practices to ensure children’s safety, (4) age-appropriate designs in task-related fMRI experiments, (5) wide differences in task performance, (6) data distortion in the presence of head motion artifacts, (7) the availability of age-appropriate atlases and customized templates for group analyses, (8) age-dependent changes in brain structure (anatomy) and hemodynamic responses, and (9) interpretation of brain activations that may change with time and expertise. Many reviews in the fields of pediatric and developmental neuroimaging have proposed very useful recommendations on how to effectively scan young participants and process data (e.g. Kotsoni et al., 2006; Church et al., 2010; Greene et al., 2016; Fassbender et al., 2017; Cusack et al., 2018; Wilke et al., 2018). In the paragraphs below, we focus on three major issues pertaining to studying learning and development in children, using fMRI.

The first issue concerns the selection and characterization of participants. As discussed below, young participants generally display higher group heterogeneity compared to typical adult populations, due to differences in age, abilities, expertise, and to familiarity with tasks and research environment. Other issues related to comorbid conditions (Margari et al., 2013; Willcutt et al., 2019), medication status, and clinical assessment need to be taken into account (Greene et al., 2016). In addition, individual differences in hemodynamic responses may vary with participant age (Kozberg and Hillman, 2016), with many studies reporting age-dependent changes in cerebral blood flow (Wu et al., 2016) that could impact on the apparent differences in brain activation between groups of variable age (Vasta et al., 2018). These age-dependent differences in hemodynamic response complicate the interpretation of brain function differences across child groups of differing age (and school year). Age-appropriate flexible response functions or model-free approaches should thus be considered (Cusack et al., 2018). For multi-subject fMRI studies in young learners, high between-subject variability is expected, so it is useful to gather as much information as possible about participants (i.e. demographic and behavioral data), as this can be valuable for optimally modeling data and interpreting results.

The second issue is head motion, which has been widely discussed in previous studies. Children’s head motion has the potential to systematically affect individual differences in BOLD changes and in measures of functional connectivity within and across groups (Satterthwaite et al., 2012; Engelhardt et al., 2017; Fassbender et al., 2017). This issue is complicated further, because in-scanner head motion also correlates with many variables of interest such as age, clinical status, cognitive ability, and symptom severity, and hence it has the potential to introduce systematic bias in brain activations and connectivity (Satterthwaite et al., 2019). Some methodological solutions for minimizing head motion have been suggested in this active domain of research, including, for example, the application of prospective motion correction techniques (Zaitsev et al., 2017), robust pre-processing tools (Esteban et al., 2019), the use of screen-printed flexible MRI receive coils for better comfort (Corea et al., 2016; Winkler et al., 2019), or size-optimized coils to increase acceleration in MRI scan acquisition in younger participants (Keil et al., 2011). Furthermore, alternative behavioral procedures used in fMRI for children have been shown to successfully reduce anxiety, improve compliance, and minimize in-scanner head motion in young children, including pre-scan training with a mock scanner (de Bie et al., 2010; Li et al., 2019b), watching an introductory video about what to expect before scanning (Szeszak et al., 2016; Waitayawinyu and Wankan, 2016), using head restraints that can be tolerated by children (Fassbender et al., 2017), communicating with participants at regular intervals between runs of the paradigm and checking that they are comfortable (Fassbender et al., 2017), watching low-demand movies during data collection of task-free fMRI data (Vanderwal et al., 2015), and providing real-time visual feedback about head movement (Greene et al., 2018). These procedures should be included in fMRI experiments when scanning school-aged participants.

The third issue is task performance, which is of concern to behavioral testing in general, but it can be more tenuous under fMRI conditions. In addition to making scanning sessions as comfortable as possible for children to maintain attention to the paradigm (Fassbender et al., 2017; Wilke et al., 2018), it is recommended to collect in-scanner performance by administrating active tasks rather than passive tasks to keep participants engaged, and to incorporate strategies that can deal with group performance differences (Church et al., 2010). To maintain engagement, the use of shorter runs is preferable to longer runs. In addition, tasks should be doable by all participants, who may differ in age and abilities; this would ensure that task demand is matched across participants. Real-time monitoring of in-scanner performance helps in ascertaining that children are engaged in the task (Wilke et al., 2018). In addition, positive reinforcement can also help in maintaining a child’s motivation to “do well” inside the scanner and improve amenability to the task and performance; these include social rewards such as frequent words of encouragement or tangible rewards. These procedures allow for the collection of high-quality artifact-free fMRI data.

### Investigating Heterogeneous Cohorts With Wide Variability in (Dis)Abilities

fMRI provides a remarkable non-invasive tool to study cognition across the lifespan and in vulnerable young populations. These populations are likely to be heterogenous due to various factors that can impact a student’s brain during the school years, for example, age- and learning-related changes (Chyl et al., 2018; Caras and Sanes, 2019; Geng et al., 2019). One important question in education is to characterize the brain factors that can explain the wide variability in learning (dis)abilities. Learning capacity and rate vary considerably across students even within the same educational setting, with some students learning quickly while others struggle with learning. For that reason, fMRI in education should pay attention to the individual effect and go beyond the typical aggregate group inferences in fMRI that assume the ‘average’ brain can fit all sizes. Although this makes it possible to link an average brain to an average behaviour, it ignores any deviation from the mean and treats it as noise. Many fMRI studies have indeed highlighted that this framework is too reductionist, and that between-subject variability is meaningful (e.g. see a recent review by Seghier and Price, 2018). Paying attention to the individual effect is useful when studying brain correlates of skills that are known to vary considerably across students, such as reading skills (for example Seghier et al., 2008; Fischer-Baum et al., 2018; Malins et al., 2018). To fully appreciate variability in brain function, rich demographic and behavioral data are needed to better model and interpret fMRI findings, including accuracy, response times, error types, and learning rates; post-scan debriefing questionnaires can also provide valuable information. These data can be highly useful when mapping functional plasticity in childhood with fMRI (Dennis et al., 2014).

Variability in brain function may result from complex genetic-by-environment interactions, and thus, may contain signatures of individual differences in abilities. For instance, previous fMRI studies have shown that variability in brain activations can be associated with individual differences in many cognitive and behavioral dimensions such as short-term memory capacity, motivational state, learning aptitude, attention shifting efficiency, cognitive flexibility, academic diligence, decision making, inhibitory efficiency during executive functions, and other higher cognitive abilities (Todd and Marois, 2005; Wager et al., 2005; Chuah et al., 2006; Locke and Braver, 2008; Barnes et al., 2014; Armbruster-Genç et al., 2016; Asaridou et al., 2016; Hilger et al., 2017; Fuhrmann et al., 2019). To maximize the usefulness of fMRI for educators, inter-individual variability in brain function should be treated as data rather than noise (Kosslyn et al., 2002; Thompson-Schill et al., 2005; Seghier and Price, 2018). In this context, accurate characterization of the variability in typical populations allows us to derive sound characterisations of the neuronal correlates of learning difficulties at the individual level. In other words, to understand what constitutes atypical processing, we must first understand what can be considered typical, by accurately estimating the typical range of variability in brain function. This issue is crucial to both diagnostic and prognostic purposes (Seghier and Price, 2018).

Furthermore, understating between-subject functional variability recognizes that a given brain function can be sustained by different processing pathways, and the activation of these pathways may vary with individual strategies and preferences (Price and Friston, 2002; Seghier et al., 2008). Individual differences in the activation of these processing pathways may yield less consistently overlapping effects in typical aggregate fMRI group statistics. However, by looking at structure or patterns in the between-subject variability, it becomes possible to decode the different processing pathways that can sustain a given function (Seghier and Price, 2009). One useful framework is to model between-subject variance in activation as a mixture of different subgroups instead of assuming one single homogeneous group: the goal is to maximize similarity within subgroups at the same time as maximizing differences between subgroups. This rationale has been used previously to tease-apart different subgroups of healthy participants who used different strategies to execute the same tasks (Kherif et al., 2009; Cerliani et al., 2017). Previous studies in clinical fMRI have shown the usefulness of understanding variability in brain function to explain inter-patient variability in deficit severity and recovery capacity after brain damage (Price et al., 2017), and ultimately, to design tailored individualized interventions and generate accurate individualized predictions (Reinkensmeyer et al., 2016). fMRI for education should embrace these emerging trends to assess meaningful individual differences in development, learning, and cognition (Brown, 2017; Foulkes and Blakemore, 2018).

In the same way, when investigating atypical processing in children with learning difficulties such as dyslexia or dyscalculia, it is useful to acknowledge that typical and atypical processing do not always reflect a categorical distinction with clear-cut thresholds (Marquand et al., 2019); but rather, lie on a continuum of the full spectrum of learning and cognitive processing. Atypical processing itself varies (e.g. different types of learning difficulties) (Gomides et al., 2018), and characterization of these sub-types rests upon a better understanding of the variability in brain function, in particular when designing intervention procedures. Accordingly, defining fMRI-based brain markers of learning difficulties at the individual level requires optimal modeling (Thomas et al., 2019b) for parsing the heterogeneity in school-aged young populations and for understanding the comorbidity between learning difficulties (Willcutt et al., 2019). Last but not least, for an individual struggling with learning in one way, neuroscience can advise on the most efficient alternative way, based on the available processing pathways in that individual. This would also be powerful for developing educational applications, because educators are interested in knowing more about the wide variability in learning (dis)abilities, with the ultimate aim of personalizing teaching methods (Immordino-Yang and Gotlieb, 2017).

### Studying the Brain Under Natural and Ecological Conditions

fMRI findings that have the best translational potential are those that can be obtained under experimental conditions that are as close as possible to ecological, or real-world, contexts (Lowe, 2012; Maguire, 2012). Observing the brain while executing time-locked pseudorandomised repeated stimuli might not be the ideal context to fully understand how the brain works in daily-life. In the last decade, some studies have looked at conducting fMRI studies while participants’ thoughts wander freely (i.e. resting-state fMRI), are in natural, or in uncontrolled stimulation conditions, such as sleep, mental reasoning, continuous reading, listening to narrative stories, watching movies, and more, (e.g. Bartels and Zeki, 2004; Malinen et al., 2007; Lahnakoski et al., 2012; Wang et al., 2015; see a recent review by Vanderwal et al., 2019). This opportunity to observe the brain in natural contexts is highly appealing to educators looking for biological explanations of mental states that could hinder learning quality, such as, mind wandering, misbehavior, poor memory retention, lack of concentration, demotivation, disinterest, and fatigue, inside the classroom. Naturalistic fMRI experiments can help to ensure that the thoughts or behaviors being investigated are not perturbed or constrained by the imaging protocol, making it possible to engage neural circuits under real-life conditions (Hasson and Honey, 2012; Maguire, 2012). For example, using naturalistic protocols, it was possible to monitor brain activity while participants were playing video games (Mathiak and Weber, 2006) or interacting in natural social scenarios (Deuse et al., 2016) using for instance hyperscanning methods (scanning more than one person simultaneously) (Wang et al., 2018). Recently, the development of reasoning skills in children aged between 3 and 12 years was mapped with fMRI during movie watching (Richardson et al., 2018). In another example, an fMRI session under natural conditions (watching/listening of natural audio-visual movie tracks) enabled researchers to look at language processing while recording eye gaze trajectories (Hanke et al., 2016). In the context of education, these unconstrained stimulations (e.g. watching video clips) have the potential to engage a wide range of brain systems given the diverse streams of information that are typically contained in movies, which can capture dynamic real-world processing; this would ultimately provide a richer depiction of brain function at the individual level (Jang et al., 2017; Moraczewski et al., 2018). Last but not least, recent studies have also shown the potential of naturalistic approaches in studying vulnerable populations, including individuals with autism spectrum disorder (Rosenblau et al., 2016).

Another widely studied topic in functional neuroimaging of the brain under natural conditions is mind wandering. This has potential applications for educators, since mind wandering has been linked to poor outcomes in a wide range of learning tasks (Smallwood et al., 2007). Moments of mind wandering tend to disrupt memory, comprehension, participation in the classroom, and intellectual functioning (Smallwood and Schooler, 2015), in particular when the external sensory stimuli become uninteresting, repetitive, and familiar. Previous resting-state fMRI studies have shown that mind wandering involves an intricate interplay between different networks, in particular the default-mode network (Raichle et al., 2001). These fMRI findings revealed the different neuronal correlates of mind wandering, which could motivate the development of strategies to minimize mind wandering at inopportune times. This includes the need to update the sensory inflow and make it less predictable in classrooms. Interestingly, some studies have investigated the possibility of controlling and modulating mind wandering using stimulations on core regions of the default mode network (Kajimura et al., 2016).

Although fMRI textbooks still consider standard laboratory-based fMRI paradigms as a better-controlled way of looking at the brain, educational neuroscientists should consider the possibility of studying brain function with fMRI using naturalistic protocols, especially, given the recent sophistication of stimuli and data analysis methods. Some research topics of interest to education can be addressed sensibly only in real-world contexts, which emphasizes the importance of looking at the brain with naturalistic approaches.

### Combining fMRI With Other Modalities for Multimodal Brain Mapping

When studying brain function, we do not have a ‘golden technique’ that addresses all the questions. There are many methods, including fMRI, each has limitations, but they can often provide complimentary information (Ugurbil, 2018). Given the multifaceted developmental changes that occur in the learner’s brain at different levels (microscopic to macroscopic) and along multiple dimensions (physiology, structure and function), combining different mapping methods can provide an accurate depiction of such changes and their relationship to cognitive and behavioral growth in the preschool years and beyond (Brown and Jernigan, 2012). Many multimodal protocols have been proposed in the literature, including the widely used combination of fMRI with EEG (Pleisch et al., 2019). Here we focus on MR-based modalities that can be acquired in the same scanning environment while the participant is lying in the scanner. Concurrently acquiring information from fMRI and additional modalities can help to quantify longitudinal changes and between-subject differences in brain function, hemodynamics, and structure (Turner and Geyer, 2014; Reid et al., 2016; Larivière et al., 2019). For example, changes in white matter tract microstructure are not directly seen by fMRI (Giorgio et al., 2010; Slater et al., 2019), so adding a diffusion MRI protocol during the same scanning session would provide an opportunity to assess white matter microstructure and use this information to explain changes in brain function (e.g. Richards et al., 2017).

Indeed, combining fMRI data with anatomy information is tremendously helpful for optimal modeling of brain function (Turner and Geyer, 2014; Higgins et al., 2018). One classic example is the development of (functional) language lateralization in school-aged children (Szaflarski et al., 2006; Groen et al., 2012; Nora et al., 2017), a question better understood if combined with information about the maturation or development of major white mater tracts such as the arcuate fasciculus (Sreedharan et al., 2015; Silva and Citterio, 2017). Recent studies have shown that atypical brain functions combined with information about alterations in brain structure explained better symptom severity in children with ADHD (Zhan et al., 2017; Wu et al., 2019). Another example concerns the evaluation of the brain correlates of math or language learning after instruction or intervention where a combination of anatomy and function information provided more accurate explanations than functional information alone (Supekar et al., 2013; Evans et al., 2015; Thieba et al., 2019), and proven to be useful in explaining the co-occurrence of reading and mathematical difficulties in children (Skeide et al., 2018).

Recent development of non-invasive MR-based protocols has opened many opportunities to provide accurate multiscale and multimodal explanations of brain-behaviour associations, including the assessment of brain perfusion with arterial spin labelling (Leung et al., 2016; Armitage et al., 2017), brain morphology (i.e. brain volume, sulci shape and depth, gray matter density, cortical thickness, myelin and ion density) with multiparametric quantitative MRI (Kim et al., 2017; Carey et al., 2018), and white matter microstructure with diffusion MRI (Lundell et al., 2019). When designing experiments with task-based fMRI paradigms in children, it is recommended if scanning time permits, to add a task-free fMRI run for resting-state network segregation, a diffusion MRI acquisition and a high-resolution anatomical scan. To manage experiment length, some of these acquisition protocols may be completed on a different visit, though acquisition time might no longer be an issue in the future with the emergence of new fast MRI acquisition schemes (LeVan et al., 2018; Polak et al., 2019). Many analysis software packages have made the processing of multimodal MRI data accessible even for non-experts.

### Multisession Scanning to Assess Developmental or Post-intervention Changes

For studying brain-behaviour associations, multisession fMRI acquisitions provide a better framework to address questions that are relevant to education, in particular for questions where age and post-instruction time have a strong impact on brain function (Evans et al., 2016; Brod et al., 2017). Thanks to plasticity, the brain changes dramatically across development and in response to experience: for example, during learning, skill acquisition, or following intervention through behavioral protocols or brain stimulation techniques. Many fMRI studies have demonstrated the possibility to detect changes in brain function and connectivity during the development of reading skills or following intervention in children with reading difficulties (Horowitz-Kraus et al., 2015; Murdaugh et al., 2015; Wise Younger et al., 2017; Smith et al., 2018; Yu et al., 2018; Nugiel et al., 2019), with the opportunity to accurately predict individual behaviour (Scheinost et al., 2019). Typically, intervention-induced time-dependent changes can be characterized in cross-sectional or longitudinal fMRI studies. The latter offer better control of potential confounds or nuisance variables, but sometimes lack statistical power and can be constrained by time and funding. In contrast, cross-sectional studies can help to increase power within a reasonable time window. Researchers and educators should be aware of the limitations of each type of design when assessing intervention-induced changes in brain function (King et al., 2018).

One particular example of multi-session fMRI is the investigation of changes in brain function after intervention with neuromodulation techniques, with the possibility to assess effects at the individual level (Abutalebi et al., 2009; Sebastian et al., 2017). One class of intervention protocols, used mainly in clinical neuroscience, is brain stimulation by transcranial direct current stimulation (tDCS) to modulate cortical excitability and hence to enhance cognition. Many studies have used tDCS to improve cognition in patients with Parkinson’s disease, Alzheimer’s disease, hemi-neglect, epilepsy, and aphasia (Flöel, 2014; Cappon et al., 2016). The application of electrical stimulations in combination with behavioral intervention can enhance recovery capacity, though studies vary considerably in patient selection, treatment-delivery protocols and outcome-measures (Cappon et al., 2016; Al Harbi et al., 2017). For educational purposes, tDCS has also the potential to facilitate learning, including improving verb learning (Fiori et al., 2018), word reading (Xue et al., 2017), working memory (Berryhill and Jones, 2012), arithmetic problem-solving (Hauser et al., 2016), and in treating children with dyslexia (Costanzo et al., 2019), and autism (Osorio and Brunoni, 2019).

Another intervention protocol comes from neurofeedback procedures (Sitaram et al., 2017) where participant-specific brain-related signal is used as feedback to train the participant in self-regulating brain function (Thibault et al., 2018). This protocol can induce brain plasticity by means of self-modulation of brain activity in real time. Specifically, fMRI-based neurofeedback protocols use real-time measures of brain activation as a feedback signal, and this signal can summarize regional brain activations, a multivariate pattern (i.e. decoded neurofeedback), or a connectivity pattern in a targeted network (i.e. connectivity-based neurofeedback); for review see (Watanabe et al., 2017). Neurofeedback protocols have been used for diverse conditions, including ADHD (Zilverstand et al., 2017; Rubia et al., 2019), motor disorders (Liew et al., 2016), and cognitive rehabilitation in stroke populations (Kober et al., 2015; Renton et al., 2017). Another interesting application of fMRI-based neurofeedback is in emotion regulation (Linhartová et al., 2019), where self-regulation of amygdala activity helped participants to improve emotion control and reduce anxiety (Herwig et al., 2019), which is highly beneficial in the educational context given the high burden of many anxiety disorders on children functioning (Schwartz et al., 2019). Before looking at the translational potential of such intervention protocols to education, their effectiveness should be assessed with randomized controlled trials and randomized controlled cross-over trials, as has been conducted for tDCS in stroke survivors (Elsner et al., 2015), tDCS for enhancing working memory capacity in healthy individuals (Ikeda et al., 2019), and neurofeedback in adults with ADHD (Schönenberg et al., 2017).

When using fMRI to assess longitudinal changes to brain function, either during learning or during the course of an intervention, it is important to appreciate the degree of reliability one can get from fMRI studies with children and the different limitations and challenges afforded by longitudinal designs (Vetter et al., 2017; Herting et al., 2018; King et al., 2018; Madhyastha et al., 2018; Telzer et al., 2018). In addition, when assessing statistical significance of longitudinal changes, it is recommended to go beyond reporting values of *p* (Halsey et al., 2015), because values of *p* provide poor information about replication (Cumming, 2008). A *p* indicates only whether a given intervention is working or making a difference, but the effect-size provides an estimate of the size of the change or difference. Estimates of effect sizes and confidence intervals (Cumming, 2014) should be used to provide better estimates of the magnitude and precision of an intervention effect or of a developmental change within or between participants. It is true however that many fMRI studies do not include effect-size estimates, and interventions are frequently selected according to significant effects only, but if the magnitude (effect-size) happens to be small, then this would explain why these interventions have shown little success in clinical or educational settings.

Other alternative methods for generating useful inferences in fMRI rely on Bayesian statistics. Bayesian analyses can be more informative and more flexible than traditional methods when it comes to hypothesis testing, model comparison and parameter estimation (Wagenmakers et al., 2016, 2018). For instance, Bayesian hypothesis testing allows researchers to estimate evidence and monitor its progression as real data are added, with the attractive possibility to take into account prior knowledge and to identify the most useful explanations (i.e. models) given the observed data (Konig and van de Schoot, 2018). Many practical tools have been introduced in the neuroimaging literature to make Bayesian approaches accessible to fMRI users interested in testing the presence of an effect and in model selection (see for example, Rosa et al., 2010; Han and Park, 2018, 2019; Soch and Allefeld, 2018). Bayesian approaches can also help in optimal modeling of imaging biomarkers that may change longitudinally (Aksman et al., 2019), which can open new opportunities to examine effects that vary with age and instruction.

### Providing System-Level Mechanistic Explanations of Brain Function

Many researchers in the field of educational neuroscience have begun to recognize that there is no single brain region or connection that is indicative of individual learning capacity (Frith, 2011). Explanations of learning must therefore be expressed at the system level and be derived from mechanistic accounts, with the ultimate aim to identify the exact brain circuitry that can sustain a given mental process (Kopell et al., 2014; Churchland and Sejnowski, 2017). Brain regions do not operate in isolation: identifying the set of interacting regions (i.e. a brain network) or networks that sustain a given task, provides a biologically-plausible way of explaining brain function and behaviour (Mišić and Sporns, 2016; Mill et al., 2017). This network approach offers a more meaningful explanation of brain function in vulnerable populations: many disorders and learning disabilities are better framed as atypicalities in brain connectivity (Du et al., 2018), including for example, autism (Yahata et al., 2016) and schizophrenia (Friston, 2002). Making inferences at the system level opens new possibilities for understanding and treating brain disorders (Thiel and Zumbansen, 2016), and in understanding brain-behaviour associations. For example, it is possible to derive useful measures or scores with task-based networks to generate individual predictions about concept knowledge in STEM learning (Cetron et al., 2019).

Within the network approach, a recent trend has been to look at brain networks during rest (Lowe, 2012). Resting-state networks are remarkably similar to the networks involved in task execution (Mennes et al., 2010; Tobyne et al., 2018), and examining resting-state networks is very useful, because this at-rest connectivity can (1) shape task-dependent connectivity, (2) reflect, albeit not equivalently, how regions are anatomically connected, and (3) provide markers or signatures related to abilities and skills (Koyama et al., 2011; Laird et al., 2011; Mennes et al., 2011; Sala-Llonch et al., 2012; McFarland, 2017; Dubois et al., 2018; Tobyne et al., 2018; Zhang et al., 2019). This intrinsic connectivity of the brain can predict task performance (Baldassarre et al., 2012), recovery pathways after brain injury (Carter et al., 2012), longitudinal changes (Farah and Horowitz-Kraus, 2019; Zhao et al., 2019), and future learning (Mattar et al., 2018). One alternative suggested in recent work is to combine both task-free and task-related connectivity to derive reliable biomarkers of learning difficulties (Elliott et al., 2019). Recent work has also shown the possibility to derive functional connectome fingerprints (i.e. ‘connectotype’) to discriminate between individuals, to accurately generate individualized predictions (Miranda-Dominguez et al., 2014; Finn et al., 2015; Li et al., 2017), and to better understand the neurobiology of learning disorders (Bailey et al., 2018). With segregated brain networks, there is also the possibility to perform statistics on connections (termed edges), using graph theory analyses (Reijneveld et al., 2007), to quantify some useful connectivity metrics (Rubinov and Sporns, 2010) that can serve to discriminate between participants, tasks, and populations (e.g. Yourganov et al., 2010; Li et al., 2014; Khazaee et al., 2015; Caeyenberghs et al., 2017; Edwards et al., 2018).

Perhaps most interestingly, mapping brain networks can help explain how the brain learns: by looking at network reshaping with age (Song et al., 2014) and learning (Bassett et al., 2011; Fatima et al., 2016; Dresler et al., 2017; Mattar et al., 2018), we are able to understand how network changes and maturation enable learning (Chan et al., 2016; Bogdanov et al., 2017). Armed with these system-level inferences, individual abilities can be predicted, as shown recently in ADHD (Rosenberg et al., 2017), and in predicting memory performance improvement after training (Dresler et al., 2017). Recent applications of this emerging network neuroscience of learning can provide unique insights into adaptive neural processes, the attainment of knowledge, and the acquisition of new skills (Bassett and Mattar, 2017; Bogdanov et al., 2017; Cetron et al., 2019; Zhang et al., 2019). Many studies have highlighted the usefulness of network analyses and the possibility to derive solid biomarkers of brain disorders (Bassett and Bullmore, 2009; Braun et al., 2018; Du et al., 2018); for example for reading difficulties (Bailey et al., 2018; Edwards et al., 2018) and autism (Hong et al., 2019). When generating explanations about both brain structure and function, it is important to keep in mind that the mapping between anatomical networks - anatomical connections, and functional networks – statistical associations between functional responses, is not necessarily a linear one-to-one mapping (Meier et al., 2016; Liang and Wang, 2017). Although both types of connectivity show some similarity, they provide complementary information about the correlates of brain disorders (He et al., 2017; McColgan et al., 2017; Vega-Pons et al., 2017), which is an important conceptual issue to keep in mind when analyzing multimodal MRI data in children.

Ideally, inferences at the network or system level should encompass mechanistic models of brain function, or how connections work together, for which models of effective connectivity are needed (Friston, 2009). There is a conceptual distinction between functional connectivity and effective connectivity. Functional connectivity represents the statistical associations between regional timeseries, but there is no information about the direction of the interactions; it establishes that connections, either mono- or poly-synaptic, are present but not their direction of action or causality. This type of connectivity is widely used to derive inferences at the network level as detailed above. In contrast, effective connectivity represents the causal, directed, influences between neurons or neuronal populations and thus, provides estimates for the direction of the effects between regions. Effective connectivity models are key to understanding how brain regions work together and interact to process information (Friston, 2011). Recent approaches at high magnetic fields allow layer-specific activations to be detected, which can ultimately estimate the directions of causation between brain areas (Turner, 2016; Huber et al., 2017). One tool that has been widely used in fMRI in adults is dynamic causal modeling (DCM) (Friston et al., 2003; Seghier et al., 2010; Razi et al., 2017), which allows us to make inferences at the neuronal level through finer modeling of neurovascular coupling (Havlicek et al., 2015) and to compare between different explanations of the same data. The output from DCM can be related to many behavioral outcomes, including classification between typical and atypical participants on an individual (Brodersen et al., 2011) or group basis (Friston et al., 2016; Zeidman et al., 2019), and explaining and predicting behaviour through modeling (Rigoux and Daunizeau, 2015). Providing mechanistic explanations can provide unprecedented understating of how the brain implement a given cognitive process, as shown recently in young children across literacy development (Morken et al., 2017).

Armed with these biologically-based mechanistic accounts, it is possible to understand how typical processing is implemented in the brain and how atypical behaviour can emerge, with the potential to define efficient intervention protocols. Mechanistic explanations of brain function are needed for future development of individualized tools and interventions in education. To illustrate this rationale, we can consider the example of an acquired skill like word reading: if we have a mechanistic model that includes the brain areas that sustain reading skills, how these areas communicate together, how behavioral manipulations (e.g. word frequency, familiarity, imageability, sensory modality) modulate the interactions between different subsystems (language, executive, attentional, memory and control), how the reading system changes with expertise, and the size of typical between-subject variability in normal function, then we should be able to make predictions about how normal reading should proceed, the optimal conditions to activate the reading system, and the alternative reading pathways and the potential interventions that can be administrated to learners who struggle with reading.

### Reporting Negative fMRI Findings

The selective publication of positive effects is a well-known bias in the neuroimaging literature that is damaging not only to the integrity of science but also to its ingenuity in solving problems (Ioannidis, 2005). This problem may lead to the perpetuation of (false) positive effects until they become erroneously accepted as fact (Nissen et al., 2016), which leads to misrepresentation or misunderstanding by the media or the general public (Gonon et al., 2011), in particular, when it involves topics of great interest to the general public, such as education and when providing a neuroscience explanation of such effects (Weisberg et al., 2008). Thus, a shift is needed to encourage the publication of null or negative results. Any translational effort to the classroom must consider the balance between what can or cannot be done and what works or does not work in brain research. Negative findings are becoming the missing piece in the neuroscience literature (Pfeffer and Olsen, 2002; Schooler, 2011; Parsey, 2018) but they are needed to derive the most unbiased of scientific practices that can help to bridge the gap between neuroscience and education. Without publication of sound and relevant negative findings, the educational neuroscience literature will be skewed, and the correction of false positives difficult; this may lead to time and resources wasted on developing ineffective teaching methods that happen to be based on skewed or false brain research findings.

It is in this context that educational fMRI should emphasize the importance of replication studies. This would help to test findings in different environments (site, scanner, sequence) and samples. Replication studies can help to explain findings that are reliable and robust, along with findings that can be explained by other confounds. Another practice that can help minimize this bias in publication of positive findings and improve transparency is preregistration (Gorgolewski and Poldrack, 2016). This entails plans and predictions being registered prior to data collection, which helps to avoid the practice of selective reporting of desirable findings based on exploratory analyses. Having a proper research data management plan will also help to improve rigor and reproducibility (Borghi and Van Gulick, 2018) when using fMRI on children. Last but not least, data sharing and data repositories can also provide another solution to this issue by making data available for validating previous reports, testing new hypotheses, or aggregating with other datasets to increase statistical power.

There are many reasons why some positive findings have not always been replicated, including differences in experimental protocols (Carp, 2012), different processing tools (Bowring et al., 2019), different statistical manipulations (Nieuwenhuis et al., 2011; Woo et al., 2014; Eklund et al., 2016), limited statistical power with small sample sizes (Button et al., 2013; Lorca-Puls et al., 2018), and high variability in fMRI responses (Bennett and Miller, 2010; Dubois and Adolphs, 2016). Another overlooked issue concerns how researchers interpret their findings, especially when it comes to attributing a given function or role to an activated brain region, using ‘reverse’ inference (Poldrack, 2006). Inconsistencies are not always about the localization of effects, but in the interpretation of their function, which is usually carried out by manipulating different comparisons between conditions (contrasts, masking, conjunctions…etc.). Before considering positive or negative results across studies, it is necessary to appraise how researchers have assigned functions or cognitive processes to specific brain regions. In sum, educators should not overlook replication studies and the many fMRI studies with negative results.

### Data Sharing and fMRI Data Repositories

The typical sample size in multi-subject task-based fMRI studies is around 16–30 participants, usually predefined arbitrarily or based on power analyses. This allows for inferences at the group level, but it might not be enough for optimal replicability (Turner et al., 2018) when dealing with small population effect sizes, heterogenous groups, individual analyses, or in testing the influence of numerous demographic and behavioral variables on brain function. In that context, data repositories and databases offer an exciting opportunity for data sharing and mining, and for testing specific hypothesis with hundreds of brain scans. This would help to ensure high statistical power (Button et al., 2013) and to generate robust neuroscience findings while taking into account the impact of many confounds (Nichols et al., 2017). There has been a call in the neuroimaging community to support data sharing, and in response, databases have been established with the ultimate aim of depicting comprehensive models of typical and atypical brain function. The gains are not only in improving reproducibility and reliability (Zuo et al., 2014), but also in devising useful models that can predict behaviour in heterogeneous populations (Mueller et al., 2005; Ofori et al., 2016; Satterthwaite et al., 2016; Thompson et al., 2017). Access to big data provides the opportunity to conquer the problem of a lack of normative data in assessing the range of brain function and anatomy, and this would open new avenues for neuroimaging research for educational applications.

Shared data can also be utilized in meta-analyses to inform future fMRI studies and motivate the development of new intervention protocols. Previous meta-analysis studies provided very useful insights about many cognitive processes (see Houde et al., 2010; Pollack et al., 2015; Han, 2017; Yaple and Arsalidou, 2018; Bottenhorn et al., 2019), with the possibility to generate summary maps with high statistical validity using for instance activation likelihood estimation (ALE). This ALE meta-analysis takes into account the spatial uncertainty due to the inter-subject and between-template variability of fMRI foci reported from different experiments. Meta-analysis in neuroimaging can take the form of image-based or coordinate-based analysis (Muller et al., 2018). For image-based meta-analysis, users can take advantage of the online OpenNeuro database that contains real task-based fMRI data (Poldrack et al., 2013) and the Neurovault database that contains unthresholded whole-brain statistical images (Gorgolewski et al., 2015). Another useful open tool is NeuroSynth (Yarkoni et al., 2011), a platform for large-scale automated synthesis of fMRI data. Armed with this tool, educators for instance can explore summary maps over thousands of studies about a concept or function of interest, which can help in generating prior knowledge or testable hypotheses for further investigations.

These neuroimaging databases have been supported by the neuroimaging community for diverse clinical applications, including research in Alzheimer disease (Mueller et al., 2005), schizophrenia (Wang et al., 2016), and Parkinson’s disease (Ofori et al., 2016). On the other hand, analogous databases on the many known learning disabilities with applications for learning and education are scarce, though some interesting initiatives exist for autism spectrum disorder (Di Martino et al., 2014; Payakachat et al., 2016), ADHD (Hoogman et al., 2019), and dyslexia (Lyytinen et al., 2015). It is also interesting for researchers in educational neuroscience to be involved in initiatives that investigate educationally-relevant neurodevelopmental questions (Gao et al., 2019; Li et al., 2019a) in order to understand healthy brain development (Satterthwaite et al., 2016), including The Baby Connectome Project (Howell et al., 2019), and the Lifespan Human Connectome Project in Aging (Bookheimer et al., 2019). Sharing data should be encouraged in educational fMRI to respond to the increasingly recognized need for transparent and reproducible neuroscience (Eickhoff et al., 2016).

## Conclusion

Functional neuroimaging protocols are evolving continuously in their sophistication and flexibility to expand the range of research questions and inference levels. Best practice in this ever-expanding field is improving, which will ultimately help to ensure transparent and reliable neuroscience evidence and to maximize the translational potential of neuroimaging findings to education. Although some of the issues discussed here are not specific to educational fMRI, any progress in them will directly impact on the translational potential of fMRI findings to education. Educators, whether interested in conducting neuroscience experiments or searching for the best and most useful neuroscience evidence, need to pay attention to those developments. This is critical when educators are looking for novel alternative intervention methods for students struggling to learn, as the field moves from standardized intervention protocols to targeted individualized intervention strategies, comprising both behavioral therapies and non-invasive brain stimulation. Educators and neuroscientists interested in educational questions should move beyond simplistic correlational approaches, and embrace these new trends of multimodal longitudinal designs, along with the use of advanced methods that can estimate causality in brain change to derive system-level mechanistic explanations of brain-behaviour associations. This will ultimately help to better understand individual differences, heterogeneity in learner profiles, and the co-occurrence of deficits and comorbidities. The issues highlighted in this succinct review are paramount to the development of optimal fMRI practices for school-aged young individuals, and to ensure that what gets pedagogically evaluated, is top-quality fMRI evidence.

## Author Contributions

MS helped in conceptualization, writing, and in original draft preparation. MS, MF, and CH helped in editing, revising, and in funding acquisition. All authors reviewed and approved the final version of the manuscript.

### Conflict of Interest

The authors declare that the research was conducted in the absence of any commercial or financial relationships that could be construed as a potential conflict of interest.
